# Nemaline myopathies: a current view

**DOI:** 10.1007/s10974-019-09519-9

**Published:** 2019-06-21

**Authors:** Caroline A. Sewry, Jenni M. Laitila, Carina Wallgren-Pettersson

**Affiliations:** 1 Dubowitz Neuromuscular Centre, UCL Institute of Child Health and Great Ormond Street Hospital, 30 Guilford Street, London, WC1N 1EH UK; 2 Wolfson Centre of Inherited Neuromuscular Disorders, RJAH Orthopaedic Hospital, Oswestry, SY10 7AG UK; 3 Folkhälsan Institute of Genetics, Folkhälsan Research Center, Helsinki, Finland; 4 Department of Medical and Clinical Genetics, Medicum, University of Helsinki, Helsinki, Finland

**Keywords:** Nemaline myopathy, Congenital myopathy, Rods, Rod bodies, Z line, Z disc, Animal models

## Abstract

Nemaline myopathies are a heterogenous group of congenital myopathies caused by de novo, dominantly or recessively inherited mutations in at least twelve genes. The genes encoding skeletal α-actin (*ACTA1*) and nebulin (*NEB*) are the commonest genetic cause. Most patients have congenital onset characterized by muscle weakness and hypotonia, but the spectrum of clinical phenotypes is broad, ranging from severe neonatal presentations to onset of a milder disorder in childhood. Most patients with adult onset have an autoimmune-related myopathy with a progressive course. The wide application of massively parallel sequencing methods is increasing the number of known causative genes and broadening the range of clinical phenotypes. Nemaline myopathies are identified by the presence of structures that are rod-like or ovoid in shape with electron microscopy, and with light microscopy stain red with the modified Gömöri trichrome technique. These rods or nemaline bodies are derived from Z lines (also known as Z discs or Z disks) and have a similar lattice structure and protein content. Their shape in patients with mutations in *KLHL40* and *LMOD3* is distinctive and can be useful for diagnosis. The number and distribution of nemaline bodies varies between fibres and different muscles but does not correlate with severity or prognosis. Additional pathological features such as caps, cores and fibre type disproportion are associated with the same genes as those known to cause the presence of rods. Animal models are advancing the understanding of the effects of various mutations in different genes and paving the way for the development of therapies, which at present only manage symptoms and are aimed at maintaining muscle strength, joint mobility, ambulation, respiration and independence in the activities of daily living.

## Introduction

Nemaline myopathies are a group of congenital myopathies defined by structures known as nemaline rods or nemaline bodies that stain red with the modified Gömöri trichrome technique (Dubowitz et al. 2013, 2019 in press). The spectrum of clinical phenotypes is wide and mutations in many genes (at least 12) are known to be associated with their presence in muscle biopsies. Additional pathological features such as cores, caps and fibre type disproportion (FTD) as well as the presence of only a few fibres with rods overlap with other congenital myopathies and challenge the classification of all congenital myopathies. Although structural features such as rods can be identified in a muscle biopsy, it is the combination of clinical, histological and genetic features that define a disease entity.

Histopathological features have a major role in directing molecular analysis (Dubowitz et al. 2013), but the increasing use of gene panels and exome sequencing is identifying novel genes and expanding clinical phenotypes associated with known genetic defects that result in the formation of rods. Nemaline rods are not specific for nemaline myopathies and may also occur at normal myotendinous junctions, in normal extra-ocular (eye) muscles, in ageing muscle and occasionally in a variety of other inherited or acquired neuromuscular and other disorders (see Vandebrouck et al. 2010). In vitro studies of cultured cells have shown that rods can result from metabolic stress by depletion of adenosine triphosphate (Vandebrouck et al. 2010). The diagnosis of nemaline myopathies relies on a multidisciplinary approach with careful clinical, pathological and genetic correlations. Muscle magnetic resonance imaging (MRI) has also become an important contributor to diagnosis and highlights specific patterns of muscle involvement associated with particular mutated genes (Jungbluth 2017).

The term ‘nemaline myopathy’ is usually applied to the group of muscle disorders presenting at birth or early childhood with hypotonia and muscle weakness, but cases of adult onset have also been reported, including those referred to as sporadic late-onset nemaline myopathy, many of which are of autoimmune origin, or associated with HIV (see Schnitzler et al. 2017). In addition, some patients may not present until adulthood, but careful enquiry and clinical examination often identifies problems in childhood, albeit mild. This article focuses on the congenital and childhood onset forms.

## What is a nemaline rod?

Nemaline rods were identified in the 1950s and 1960s in muscle biopsies from children with hypotonia (Conen et al. 1963; Shy et al. 1963; Schnell et al. 2000). Conen et al. (1963) described the appearance of rod-like structures in the biopsy of a child with hypotonia as ‘myogranules’ which would now be described as rod bodies. The term ‘nemaline myopathy’ was suggested by Shy and co-workers after the Greek word for thread, *nema*, as it was not clear whether the structures were separate rod-like structures or an undulating thread-like structure.

Rods stain red with the modified Gömöri trichrome technique but electron microscopy may be needed to distinguish them from mitochondria, which also stain red, especially in very small muscle fibres in neonates. Rods are considered to be derived from Z lines as they can show continuity with Z lines; they have a similar lattice structure and express similar proteins, including α-actinin, actin, tropomyosin, myotilin, γ-filamin, cofilin-2, telethonin and nebulin. Desmin is not present in the rods themselves but may be observed at their periphery. In some fibres in human muscle biopsies and in some animal models, the rod-like structures may appear as an integral part of the sarcomere, and as thickened Z lines compared with the normal width, which is usually fixed, according to the muscle fibre type (Luther 2009).

It is not yet clear how rods form, but myofibrillar rearrangement is considered to result in several abnormalities of the Z lines (Yu et al. 2004). Rods can be a secondary response to metabolic stress. Numerous rods were observed in a few patients with Complex I deficiency (Lamont et al. 2004) and in both muscle and non-muscle cells in vitro, they can be induced by a variety of substances that cause energy shortage, including ATP depletion and heat shock proteins (Vandebrouck et al. 2010). This in vitro study suggested that rods formed under different conditions vary with regard to their cofilin and α-actinin content. In addition, specific *ACTA1* mutations affected the localization of rods (nuclear versus cytoplasmic).

## Genetics

Nemaline myopathy may be caused by mutations in at least 12 genes (Table 1) and some cases are still molecularly unresolved. A recently identified gene is *TNNT3*, the gene encoding fast skeletal troponin 3 (Sandaradura et al. 2018). In addition, a homozygous mutation in *MYO18B*, encoding an unconventional myosin, has been reported as a possible cause of nemaline myopathy in an atypical case (Malfatti et al. 2015). Nemaline rods were also observed in association with a mutation in *MYO18B* but the patient also had Klippel–Feil anomaly, dysmorphic features, microcephaly and short stature (Alazami et al. 2015). Furthermore, in experimental models, myofibre assembly failed in a way not characteristic of nemaline myopathy biopsies (Berger et al. 2017; Gurung et al. 2017) but animal models often do not recapitulate human diseases precisely.

**Table 1:** Genetic causes of nemaline myopathies

Gene	Inheritance	Associated features in addition to cytoplasmic nemaline rods
*ACTA1*	de novo AD, AR, AD	Actin accumulation, nuclear rods, cores, cores + rods, zebra bodies, FTD
*NEB*	AR, (AD)	Distal myopathy, cores + rods, FTD
*TPM2*	AD, de novo AD, (AR)	FTD, caps, distal arthrogryposis, Escobar syndrome
*TPM3*	AD, de novo AD, AR	FTD, caps
*KBTBD13*	AD	Slow movement, cores + rods
*CFL*-*2*	AR	Ophthalmoplegia, cores, actin accumulation
*KLHL40*	AR	Ophthalmoplegia, rectangular rods with fringes
*KLHL41*	AR	No cores, typical pathology
*LMOD3*	AR	Rectangular rods with fringes
*MYPN*	AR	Cardiomyopathy, nuclear rods, caps
*TNNT1*	AR, (AD)	Excess connective tissue, contractures
*TNNT3*	AR	Arthrogryposis, excess connective tissue
*MYO18B* ^a^	AR	Cardiomyopathy, dysmorphism, Klippel–Feil anomaly

*AR* autosomal recessive, *AD* autosomal dominant, *FTD* fibre type disproportion

^a^*MYOD18B* is not yet clearly identified as a ‘nemaline myopathy’ as it was associated with complex phenotypes, not typical of nemaline myopathy

Other genes are also associated with the presence of nemaline rods or cap-like areas, but additional structural and clinical features are present in these patients and thus, they do not fulfil the criteria of nemaline myopathy as outlined at a European Neuromuscular Centre workshop (Wallgren-Pettersson et al. 1998), although publications may refer to them as such. For example *RYR1* and *TTN*, encoding the ryanodine receptor 1 and titin, respectively, (Sewry and Wallgren-Pettersson 2017; Oates et al. 2018), *EXOSC3* that encodes a component of the human RNA exosome complex (Pinto et al. 2019), *PPA2* that encodes the mitochondrial pyrophosphatase (Guimier et al. 2016), and *RYR3* encoding the ryanodine receptor 3 (Nilipour et al. 2018).

The commonest forms of nemaline myopathy are caused by mutations in the genes encoding skeletal muscle α-actin (*ACTA1*) and nebulin (*NEB*). Several of the other causative genes have only been shown to be mutated in a few families each, although the wide application of novel gene sequencing methods is increasing the number of patients with verified genetic diagnoses (Wallgren-Pettersson et al. 2011; Malfatti and Romero 2016). Most of the *ACTA1* mutations are heterozygous dominantly inherited mutations, often arising de novo. *NEB* mutations are usually recessively inherited, but recently, the first dominantly inherited mutation was identified in *NEB*, causing a distal form of nemaline myopathy (Kiiski et al. 2019). A great number of different mutations have been identified in these two genes (Sparrow et al. 2003; Feng and Marston 2009; Nowak et al. 2013; Lehtokari et al. 2014; Moreno et al. 2017).

Nine of the genes for nemaline myopathy encode proteins of the sarcomere, in addition, *MYO18B* is also a sarcomeric protein localized to the Z lines (Ajima et al. 2008) and may yet prove to be a causative gene of nemaline myopathy, although the phenotype of affected patients is different from others reported. The other three genes encode Kelch-like proteins, a large family of proteins possibly associated with thin filament regulation (Wallgren-Pettersson et al. 2011; Gupta and Beggs 2014; Malfatti and Romero 2016).

Nemaline myopathies occur all over the world. Some mutations have arisen as founder mutations, such as the deletion of the entire exon 55 of *NEB* in persons of Ashkenazi Jewish ancestry, with a world-wide distribution (Anderson et al. 2004; Lehtokari et al. 2009), and the *TNNT1* mutation (E180X in exon 11 causing a stop codon) in the Amish population (Johnston et al. 2000). Probable founder mutations have also been identified in *TPM3* (deletion of the first nucleotide of the last exon, c.913delA) in the Turkish population (Lehtokari et al. 2008), *KLHL40* (c.1582G>A) in the Japanese, Kurdish and Turkish populations (Ravenscroft et al. 2013), *ACTA1* (p.Asp181fsX10) in the Pakistani population (Nowak et al. 2007), *KBTBD13* (c.1222C>T) in the Low Countries of The Netherlands and Belgium (Sambuughin et al. 2010), three mutations in *NEB* (p.Ser6366Ile in ex122, p.Thr7382Pro in ex151, and p.Thr6350Profs*4 in ex122) in the Finnish population (Lehtokari et al. 2014) and in *LMOD3* (c.1648c>T) in German and Austrian populations (Schatz et al. 2018).

Currently, many mutations are identified by screening panels of genes known to be associated with nemaline myopathy. There is often difficulty, however, with mutation detection, especially in *NEB*. It is an extremely large gene (183 exons), giving rise to many isoforms in both skeletal muscle and brain (Laitila et al. 2012). The gene has multiple splice sites and a triplicate repeat region, where the most common large variants of the gene are found (Kiiski et al. 2016). Identifying both mutations in *NEB* in a patient may also be difficult because most patients have two private mutations anywhere along the length of the gene, and some of them are easily missed using massive parallel sequencing methods (Kiiski et al. 2016). Determining the pathogenicity of especially missense variants, which are numerous in *NEB*, constitutes a further diagnostic challenge. Recent advances include a targeted array which, in many patients, has helped to identify the second mutation (Kiiski et al. 2016; Zenagui et al. 2018), and also the development of functional assays for testing the effects on the protein of missense variants. For example, a nebulin in super repeat panel reveals stronger actin binding toward the ends of the super repeat region (Laitila et al. 2019), and how disease-causing mutations in *NEB* alter interactions with actin and tropomyosin (Marttila et al. 2014).

Although mutations in *ACTA1* are often dominant (and de novo, although sometimes inherited), a few rare instances of recessive transmission have also been reported (Nowak et al. 2013). Some of these recessive mutations are null mutations that result in no production of skeletal actin protein whilst in others it is present (Nowak et al. 2007; O’Grady et al. 2015).

## Clinical features

The spectrum of clinical phenotypes of nemaline myopathies is wide, even in individuals with mutations in the same gene, or in the same family. It ranges from neonates with severe disease and onset in utero, sometimes with fetal akinesia, to mild childhood-onset forms (Colombo et al. 2015; Jungbluth et al. 2018).

### Classification of nemaline myopathies

A clinical classification, mainly designed for gene discovery, was defined at an ENMC workshop in 1999 (Table 2; Wallgren-Pettersson and Laing 2000). Since then, altogether twelve genes and numerous mutations have been identified, and it has turned out that genotype–phenotype correlations are weak, or few and far between. Thus, we propose a revised, simplified classification, based on current knowledge of the spectrum of identified patients with nemaline myopathy (Table 3).

**Table 2:** Current classification of nemaline myopathies

Severe nemaline myopathy	
Intermediate nemaline myopathy	
Typical (mainstream, classical) congenital nemaline myopathy	
Mild (childhood or juvenile) onset form	
Adult-onset forms	
”Other” (unusual) forms

**Table 3:** New proposed classification of nemaline myopathies

Severe nemaline myopathy (with contractures or fractures at birth, or with no respiratory effort or no movements at birth) (*ACTA1*, *NEB*, *LMOD*-*3*, *KLHL40*, *KLHL41*, *TNNT3*, *TPM2*, *TPM3*)	
Congenital nemaline myopathy (with perinatal onset and milestones delayed but reached) (*NEB*, *ACTA1*, *CFL*-*2*, *TPM2*)	
Mild (childhood or juvenile onset) nemaline myopathy (*ACTA1*, *NEB*, *TPM2*, *TPM3*, *KBTBD13*, *MYPN*, dominant mutations in*TNNT1*)	
Recessive *TNNT1* (Amish) nemaline myopathy	
Childhood-onset nemaline myopathy with slowness of movements and core-rod histology (*KBTBD13*)	

Among the categories in Table 2, the intermediate form was designated because there was a difference between typical (mainstream) nemaline myopathy and this group of patients, in that their course of the disease was more severe. This was exemplified by the use of a wheelchair from an earlier age than may occur in the typical form, where a wheelchair, if needed at all, is often only used from the pre-pubertal growth spurt. In other words, it was only possible to distinguish this category of patient in late childhood. It has turned out that no specific “intermediate” genes have been identified; the most common genes, *NEB* and *ACTA1*, may both cause this form. To our knowledge, there are few if any definite adult-onset cases with a proven genetic cause. The rapidly progressive adult-onset form (SLONM) is often immune-mediated and responsive to treatment (see Schnitzler et al. 2017).

Since the current classification was established, additional novel clinical forms have been described, not fitting into the current classification. Examples of this are the “Amish” form caused by mutations in *TNNT1* with contractures, tremor and a progressive course (Johnston et al. 2000; Fox et al. 2018), and the form with slowness of movements and core-rod histology (Gommans et al. 2002; Sambuughin et al. 2010; de Winter and Ottenheijm 2017). There have also been publications describing patients with an unusual distribution of weakness, such as scapuloperoneal or distal weakness, or distal arthrogryposis, and it can be argued that these should be classified as separate entities. However, most forms of nemaline myopathy have individual variation in the distribution of weakness, and a number of patients may have distal contractures early or late in the course of the disease, so that lines of division are difficult to draw between such “novel” entities and the forms already described. Thus, we argue that it is time for a new and simplified classification of nemaline myopathy, which would be useful for yielding at least an estimate of prognosis in an individual patient (Table 3).

The prognosis in the severe form is often grave, but there have been exceptions, where patients have shown improvement over time (Roig et al. 1987). The typical form often follows a static or only slowly progressive course, and some patients have shown improvement, e.g. related to active physical training. Onset in childhood or at juvenile age often implies a mild course, while recessively inherited *TNNT1* (Amish) nemaline myopathy follows a relentlessly progressive course, with thoracic immobility, restrictive lung disease and often death in childhood (Johnston et al. 2000; Fox et al. 2018). The dominantly inherited form with slowness appears to follow a milder course.

Although most patients present with muscle hypotonia, there are rare patients with muscle hypertonia and a stiff gait (Marttila et al. 2014; Davidson et al. 2013), thought to stem from higher than normal calcium sensitivity (Jain et al. 2012; Donkervoort et al. 2015; Marston 2018). Muscle weakness is usually generalised, with involvement of the neck flexors, the face and proximal muscles, often with a later, additional distal involvement. Distal weakness is a particular presentation in some patients with mutations in *NEB*, but rods may not always be a present in their muscle biopsies (Wallgren-Pettersson et al. 2007). Weakness of respiratory muscles is common and an important clinical feature to monitor regularly and manage according to international guidelines (Wallgren-Pettersson et al. 2011). Insidious onset of hypoventilation is the greatest risk for this group of patients and the most common cause of death. Thus, in the absence of expert monitoring of respiratory function, respiratory insufficiency may ensue suddenly and without any preceding symptoms (Wallgren-Pettersson et al. 2004). Extraocular muscles are usually spared, except in patients with mutations in *KLHL40* and *LMOD3*, who may have ophthalmoplegia (Ravenscroft et al. 2013; Yuen et al. 2014). Cardiac involvement is rare in patients with nemaline myopathy, but has been identified in a few patients with defects in *ACTA1*, *MYPN* or *MYO18B* (D’Amico et al. 2006; Kim et al. 2011; Finsterer and Stollberger 2015; Malfatti et al. 2015; Miyatake et al. 2017). Mutations in *TNNT1* encoding troponin T were first identified in homozygous form in the Amish population, but a few patients of Dutch descent, and others of non-Amish origin have also been described (van der Pol et al. 2014; Abdulhaq et al. 2016). Characteristics are tremor and severe progressive contractures, muscle weakness and atrophy with stiffness and thoracic rigidity. A dominantly inherited *TNNT1* mutation causing a different clinical picture has been reported, with similarities to the childhood onset form caused by mutations in other genes (Konersman et al. 2017). A severe clinical picture has been described in the patient with a homozygous *TNNT3* mutation, with contractures, hip dislocation (unusual in nemaline myopathy) and ventilator dependence until death at the age of 8 months (Sandaradura et al. 2018). Patients with mutation of *KBTDB13* often have an unusual slowness of muscle movements due to slow relaxation kinetics, and cores as well as rods (Gommans et al. 2003; Sambuughin et al. 2010; de Winter and Ottenheijm 2017). In all forms of nemaline myopathies, creatine kinase levels are usually normal or only slightly elevated.

## Histopathology

**Fig. 1:**
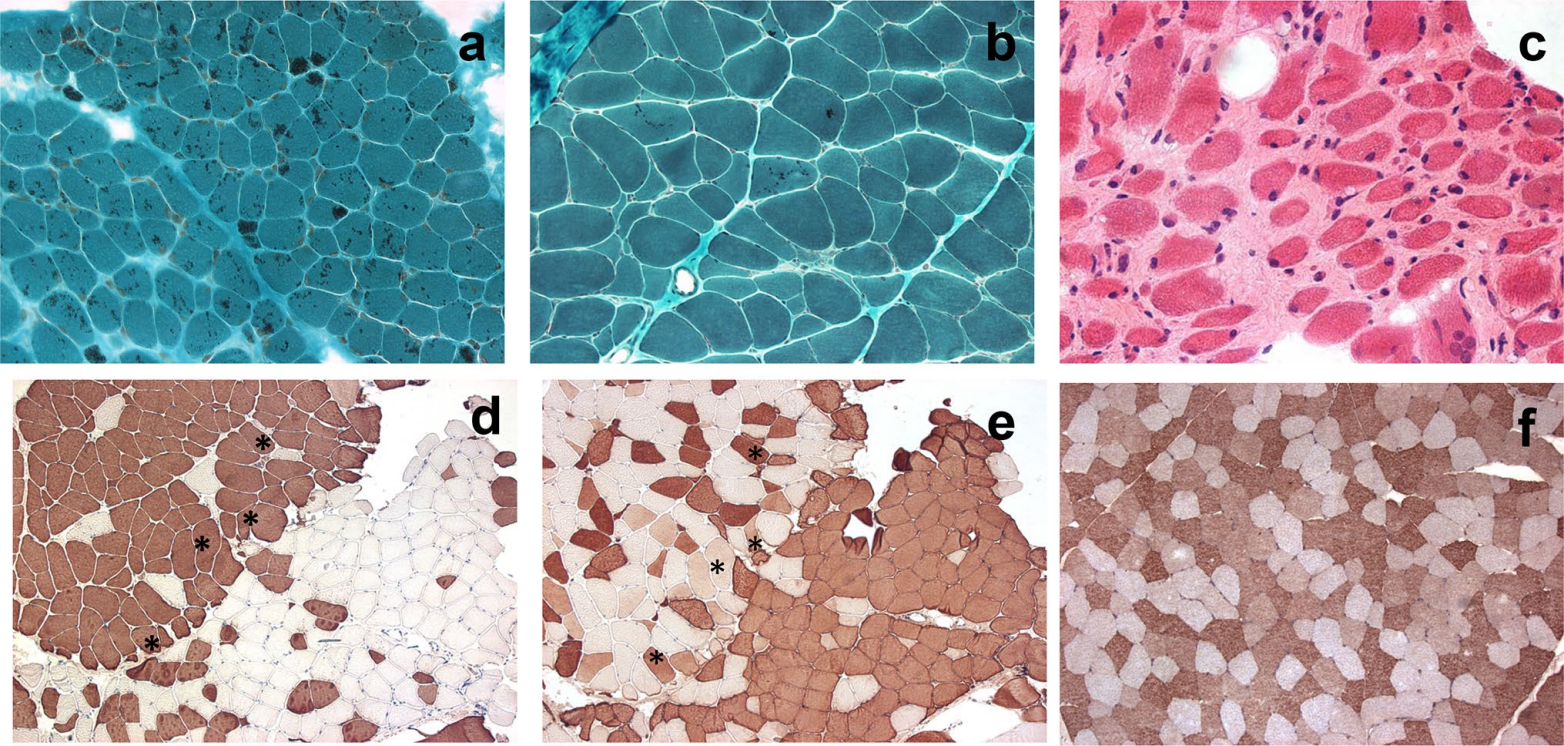
Muscle biopsies from patients with **a** a mutation in *ACTA1* (Gӧmӧri trichrome), **b** heterozygous mutations in *NEB* (Gӧmӧri trichrome), **c** heterozygous mutations in *TNNT1* (haematoxylin and eosin), **d**, **e** heterozygous mutations in *NEB* (slow and fast myosin respectively), **f** control with no molecular defects causing a neuromuscular disorder (antibody to exon 143 of *NEB*). Note the variable number and distribution of nemaline rods in a and b, the pronounced connective tissue in c, the uneven distribution of fibre types in d and e with several fibres co-expressing both isoforms (*) and the three intensities of labelling of exon 143 of nebulin in f (most of the darker fibres express fast myosin)

The characteristic cytoplasmic nemaline rods that stain red with the Gömöri trichrome technique in nemaline myopathies are usually numerous but the number and distribution per fibre can be variable (Fig. 1). The number also varies between muscles and there is no apparent correlation between clinical severity and the number of rods. Rods may be present in peripheral clusters, often near nuclei, or may be diffusely distributed, or in lines within fibres (Fig. 1). They are usually not observed in intrafusal fibres of spindles. Rods need to be distinguished from other structures that also stain red with the Gömöri trichrome technique, such as mitochondria and cytoplasmic bodies. In biopsies with very small fibres, rods may only be apparent with very high power optics. Examination of resin sections stained with toluidine blue or with electron microscopy is then helpful. An occasional cytoplasmic body may be observed in a few fibres but in three severely affected patients with the same missense *ACTA1* mutation (p.Asn94Lys), only dense accumulation of material reported as ‘suggestive of cytoplasmic bodies’ were seen, but no rods (Donkervoort et al. 2017). In addition to these patients, rods have not been observed in other patients, although mutations in genes that cause nemaline myopathy may be present. Repeat biopsies from a series of patients with *NEB* mutations and a distal myopathy did not reveal rods (Wallgren-Pettersson et al. 2007). Moreover, a family with progressive scapuloperoneal and distal weakness with an *ACTA1* mutation had no rods visible with light or electron microscopy (Zukosky et al. 2015). Mutations in *ACTA1* have also been found in cases with no rods but only cores (Kaindl et al. 2004). Sampling and variable distribution may also influence the detection of rods, for example rods may only be observed in the sample taken for electron microscopy.

Abnormal variation in fibre size is often present and type 1 atrophy or hypotrophy (fibres that have never attained normal dimensions) is common. Atrophy can be distinguished from hypotrophy by the presence of redundant basal lamina associated with atrophic fibres, using electron microscopy. There may also be hypertrophy of type 2 (fast myosin) fibres. This size variation may appear as FTD in which type 1 fibres are 12–25% smaller than type 2 and there are no additional pathological abnormalities such as rods or cores or central nuclei. FTD without structural defects can be associated with defects in several genes that cause a congenital myopathy, including several that are responsible for a nemaline myopathy (see Table 1) (Clarke 2011). Type 1 predominance, a common feature in congenital myopathies, is often present, but is not universal (Malfatti et al. 2014). Antibodies to myosin isoforms may show co-expression of slow and fast isoforms in some fibres, and there is often a variable number of fibres with foetal myosin. In addition, recent studies of a few cases caused by *NEB* mutations have revealed a predominance of fibres with fast myosin and uneven distribution of fibre types in others (Fig. 1). Involvement of type 2/fast fibres is also seen in patients with mutations in the *TNNT3* gene encoding the troponin T isoform of fast fibres (Sandaradura et al. 2018).

Immunohistochemistry of nebulin does not show a total absence of protein, although an absence has been reported in rare patients which was dependent on the mutation and the antibody used (Sewry et al. 2001; Wallgren-Pettersson et al. 2002). Recent studies with antibodies specific to exons 143 and 144 of nebulin, that are differentially spliced, suggest that there is developmental regulation of these two exons, and that exon 143 appears later in myogenesis (Lam et al. 2018). In addition, there is a clear fibre typing pattern with the antibody to exon 143 that is highly expressed in fibres with fast myosin (Fig. 1).

Rods are restricted to type 1 fibres in patients with *TPM3* mutations, as the protein is only expressed in these fibres, and in patients expressing no *TNNT3* they were reported to be restricted to type 2 fibres (Sandaradura et al. 2018), but in most biopsies they are seen in both fibre types. Rods in most biopsies of nemaline myopathy patients are present in the cytoplasm but in some cases electron microscopy reveals both nuclear and cytoplasmic rods, or very occasionally only nuclear rods (Hutchinson et al. 2006; Koy et al. 2007; Miyatake et al. 2017).

Areas with rods are often devoid of mitochondria, thus they may appear as core-like areas devoid of oxidative enzyme staining. Caution in interpretation is then needed. Sometimes cores and rods, however, may be in separate fibres or the core-like area lacking oxidative enzymes may be more extensive than the area with rods (Dubowitz et al. 2013; Scoto et al. 2013). Muscle biopsies from some nemaline myopathy patients show both rods and cores with disrupted myofibrils (Jungbluth et al. 2001; Agrawal et al. 2007; Romero et al. 2009; Dubowitz et al. 2013). Some of these patients have been classified as having a ‘core-rod myopathy’ (Hernandez-Lain et al. 2011), but they emphasise the overlapping pathological and clinical spectra of the nemaline myopathies and other congenital myopathies. Similarly, cap-like structures are regarded as being part of the histopathological spectrum of nemaline myopathies and not forming a distinct clinical entity. Although focal peripheral cap areas with myofibrillar disruption and thickened Z lines are associated with defects in the *TPM2* and *TPM3* genes (Marttila et al. 2014), they have also been described in association with defects in other genes associated with nemaline rods, *ACTA1*, *NEB* and *MYPN.* Both cap-like areas and rods can be present in the same sample and are part of the spectrum of Z-line abnormalities in nemaline myopathies (Malfatti et al. 2013).

Muscle fibre necrosis and regeneration are not usually features of nemaline myopathy. Similarly, fibrosis is rarely seen but can occasionally be extensive, for example in patients with mutations in the *TNNT1* gene (Fig. 1).

It is rarely possible to identify the defective gene from histopathological features and few of them are specific. Areas of accumulation of actin filaments suggest *ACTA1* as the cause, although such accumulation has also been seen in the rare patients with *CFL*-*2* mutations and in an animal model for this gene defect (Agrawal et al. 2007; Gurniak et al. 2014). Nuclear rods can occur in nemaline patients with *ACTA1* mutations but they have also been observed in the rare cases of *MYPN* mutation and in patients with a myofibrillar myopathy, although the clinical phenotype of these is not that of a congenital myopathy (Dominguez Rubio et al. 2016; Miyatake et al. 2017).

**Fig. 2:**
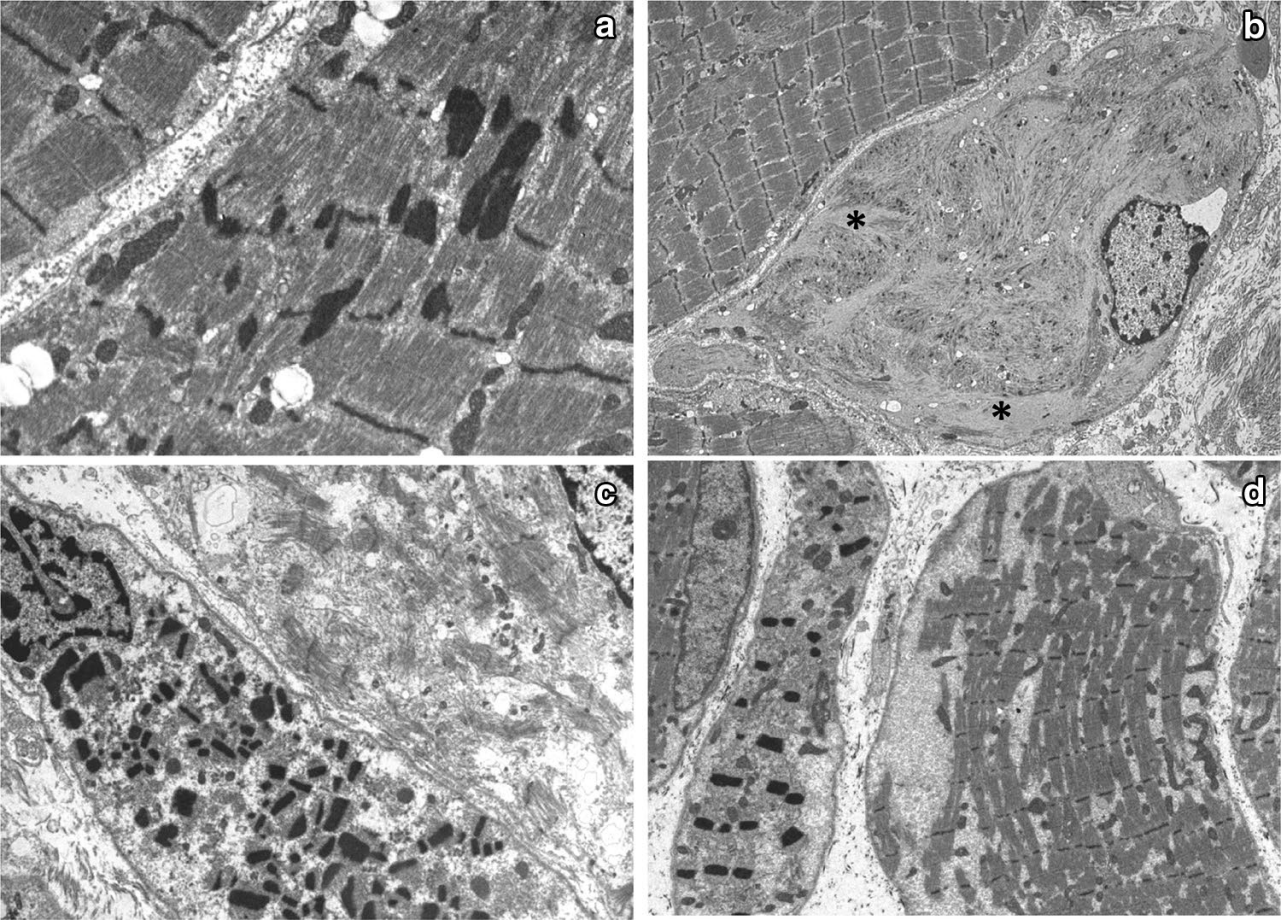
Electron micrographs of muscle biopsies from patients with nemaline myopathy caused by **a** a mutation in *ACTA1*, **b** homozygous mutation in *CFL2*, **c** heterozygous mutations in *KLHL40* and **d***LMOD3.* Note in **a** the variable size of the nemaline rods and irregularities of the Z line, in **b** the very small rods and accumulation of thin actin filaments (*) and in **c** and **d** the similar rectangular shape of the rods and the fringe-like filaments attached to many of them

All fibres expressing cardiac actin without skeletal actin is a phenomenon seen in rare patients homozygous for *ACTA1* null mutations (Nowak et al. 2007) but other patients with recessively inherited *ACTA1* mutations retain skeletal actin (O’Grady et al. 2015). Zebra bodies are also part of the *ACTA1* pathological spectrum and observed in *ACTA1* null patients (Nowak et al. 2007). They defined the original case of ‘zebra body myopathy’ in whom an *ACTA1* mutation has been identified (Sewry et al. 2015). They are not specific to *ACTA1* nemaline myopathy, as occasional zebra bodies have been observed in a variety of disorders. Electron microscopy of biopsies from patients with *KLHL40* mutations shows not only typical rods but also fibres with numerous small, rectangular rods in fibres with very few myofibrils (Fig. 2). However, patients with *LMOD3* mutations can also show similar rods (Fig. 2; Malfatti and Romero 2016). *LMOD3* biopsies have been reported to show rods with a fringe of myofibrils attached (Yuen et al. 2014), but we have observed similar rods associated with other nemaline myopathy gene mutations, in particular *KLHL40* (Fig. 2). Pairs of rods connected by thin filaments were also present in a patient with a homozygous *LMOD3* mutation (Michael et al. 2019).

## Animal and in vitro models of nemaline myopathy

To increase the understanding of the pathogenetic mechanisms leading to nemaline myopathy, several avenues of research have been opened. Animal and in vitro models of nemaline myopathy, in particular mouse and zebra fish models are being explored (Table 4). Moreover, functional studies are being performed in relation to specific gene mutations and their proteins, in vitro contraction studies of muscle fibres in relation to altered actin and tropomyosin molecules has identified differences in calcium sensitivity as a mechanism by which disruption of sarcomeric proteins leads to muscle weakness (Marston et al. 2013; Chan et al. 2016; de Winter and Ottenheijm 2017). Efforts are concentrating on the most commonly mutated genes causing nemaline myopathy, *ACTA1* and *NEB*, and descriptions have been published of a variety of knock-in and knock-out mouse models of causative mutations in these genes (Nowak et al. 2013; de Winter and Ottenheijm 2017). In addition, there are also mouse models of the more rarely affected genes *CFL*-*2*, *TPM2*, *TPM3*, *TNNT1*, *KLHL40*, *KLHL41*, *LMOD3* (see below).

**Table 4:** Mouse models of nemaline myopathies

NM mouse model (publication)	Severity	Fibre type changes	Nemaline rods/Z-line features	In vitro force	Additional features
*Neb*-KO (Bang et al. 2006)	Severe/lethal able to breath and move but possibly unable to suck	Normal	Rods, thickened/fragmented Z lines	Excitation/contraction coupling not significantly altered. Stress generated < 50% of the wild-type	Actin filaments assembled in absence of nebulin, disassemble in contracting muscle
*Neb*-KO (Witt et al. 2006)	Severe/lethal Stiff gait, kyphosis, progressive muscle weakness from days 10–20	(NR)	Rods, thickened Z lines, loss of myopalladin from Z line, abnormal Ca^2+^ homeostasis	Maximal active tension reduced. Overlap between thin and thick filaments 50% of maximum (result of shorter thin filaments). Ca^2+^ dependent recruitment of cross-bridges perturbed	Shortened thin filament length Upregulation of sarcolipin, S100A4/A9, desmoplakin, CARP/ankrd2 Tmod1 reduced and translocated towards Z line
Het *Neb*-KO (Gineste et al. 2013a, b)	No observable phenotype in vivo, mild phenotype in vitro	–	–	Maximal force production reduced by around 16% in isolated muscle (related to shift toward slower proteomic phenotype)	Shift toward a slower proteomic phenotype No impaired energy metabolism
*Neb*-cKO (Li et al. 2015)	Severe, appear normal at birth	Fibre type switching toward oxidative types, small 2B fibres	Rods, irregular, wavy and thickened Z lines	Large deficit in specific force, stiffness reduced, tension cost increased, lower number of force-generating cross-bridges	Hypotrophy of muscles rich in glycolytic fibres, hypertrophy of muscles rich in oxidative fibres KLHL40 increased
*Neb*^Δex55^ (Ottenheijm et al. 2013)	Severe. Shortened lifespan, respiratory failure possibly caused by severe diaphragm weakness	Diaphragm and EDL: decrease in type 2B fibres, increase in type 2A(X). (Joureau et al. 2016)	Rods seen with EM, at the position of the Z line	Maximal force generating capacity reduced, changes in cross-bridge cycling kinetics (reduced number of bound cross-bridges), and reduction of Ca^2+^ sensitivity of force generation. ktr lower, tension cost higher	Shortened thin filaments
*Neb*^ΔSH3^ (Yamamoto et al. 2013)	No observable phenotype in vivo, mild phenotype in vitro	–	Normal	Slightly altered force–frequency relationship. Slightly blunted sensitivity to electrical stimulation but only in a narrow range of frequencies	No structural or histological skeletal muscle abnormalities and no changes in gene expression or localization of interaction partners of the nebulin SH3 domain
*Neb*^Δ163−165^ (Li et al. 2019)	Mild to moderate	Significant shift toward slower fibre types	SOL: decrease in Z-line width; EDL: large increase in Z-line width. Loss of nebulin C-terminus may influence Z-line width to a small degree	EDL displayed a drastic loss of force, force loss in the SOL subtle	Loss of C-terminus may affect nebulin stability but length of thin filaments retained Myotilin and KLHL41 increased
*Neb*^Y2303H,Y935X^ (Laitila et al. submitted)	Mild to moderate	Smaller fast fibres in EDL, all fibres smaller in SOL	Rods predominantly in 2B fibres, thickened Z lines	Reduced specific force (single fibre level)	Core-like areas
*Acta1*-KO (Crawford et al. 2002)	Severe/lethal normal at birth and can breathe	No effect on fibre numbers, only size	No rods, normal	Lower force. Hemizygous mice produce intermediate levels of force	Lack of a haploinsufficiency phenotype reinforces that actin-based myopathies of both skeletal muscle and the heart result from functional effects caused by the mutant actin (gain-of-function mutations).
*Acta1*^H40T^ (Nguyen et al. 2011)	Severe, early lethality, males more severe than females, decreased mobility and forearm grip	Fibre atrophy and increase in slow fibres 2B fibres atrophic in EDL but 2A hypertrophied in diaphragm (Lindqvist et al. 2013)	Cytoplasmic and intranuclear rods, Z-line streaming and widened. Rods in cardiomyocytes (Lindqvist et al. 2013)	Muscle weakness associated with an improved resistance to fatigue (+ 40%) and an increased energy cost (Gineste et al. 2013a, b)	Distinct eye and facial phenotypes; accumulation of thick and thin filaments
Tg(*ACTA1*)^D286G^ (Ravenscroft et al. 2011a)	Mild to moderate, less active	(NR)	Rods, thickened Z lines, myofibrillar disruption	Weakness in both isolated muscles and single muscle fibres. Less sensitive to Ca^2+^ Smaller number of myosin cross-bridges strongly bound to actin monomers (Ochala et al. 2012)	High αB-crystallin and desmin in some fibres
Tg(*ACTA1*)^*D286G*+*/*+^*.Acta1*^+/−^ (Ravenscroft et al. 2011a)	Severe	Large variations in myofibre size	Rods, Z-line fragmentation and streaming	(NR)	Increasing mutant protein load from 25 to 45% changes mild phenotype to severe. Actin accumulation
Tg(*ACTA1*)^D286G^-EGFP(Ravenscroft et al. 2011b)	Mild to moderate, less active	Fibre type composition and fibre sizes altered	Rods, rods attached to Z line, thickened Z lines	Significantly weaker than wild-type muscle at 4 weeks of age	Core-like areas, ring fibres (2B fibres) common, internal nuclei and myofibrillar disruptions
Tg(*TPM3*)^M9R^ (Corbett et al. 2001)	Mild, late onset	Increase in slow fibres, compensatory hypertrophy of 2B and 2X fibres	Rods in clusters and areas surrounding the Z line, Z-line streaming and disruption of the sarcomeric register	Differences in normalized force. No changes in the Ca^2+^ parameters. Lower force-generating capacity caused by decrease in force per cross-bridge rather than reduction in the number of cross-bridges (Gineste et al. 2014)	Cytoplasmic bodies Reversible muscle weakness: endurance exercise alleviated muscle weakness and reduced the number of nemaline rods (Joya et al. 2004). In vivo muscle improvement not associated with changes in muscle volume or energy metabolism (Gineste et al. 2014)
*Tnnt1*-KD (Feng et al. 2009)	(NR)	Increase in fast fibres, atrophy and decrease in number of slow fibres	(NR)	Increased myofilament fatigability	Only diaphragm studied. Muscle atrophy
*Tnnt1*-KD (Wei et al. 2014)	(NR)	Atrophy and loss of slow fibres. Compensatory hypertrophy of fast fibres	(NR)	Force production lower at low stimulation frequency (40 Hz). Decreased fatigue tolerance and impaired recovery	Fast TnT increased by 60% Increase in number of type1 characterized by small size and central nuclei
*Tnnt1*-KO (Wei et al. 2014)	(NR)	Atrophy and loss of type 1/slow fibres. Compensatory hypertrophic growth of fast fibres	(NR)	Normalized force decreased by 25%. Force production lower at low stimulation frequency (40 Hz). Decreased fatigue tolerance and impaired recovery	Significant number of small type 1 with central nuclei
*Cfl*-*2*-KO (Agrawal et al. 2012)	Severe weakness, little movement, possibly unable to suck	Elevated numbers of slow fibres, fibre size disproportion	Rods seen with EM, severe sarcomeric disruptions	(NR)	Accumulation of α-actin, α-actinin-2 and TPM Fast-fibre specific genes downregulated, slow fibre genes upregulated Mitochondrial abnormalities and internal nuclei. Muscle pathology differed from nemaline myopathy, but showed combined features of actin-associated myopathy and myofibrillar myopathy
*Lmod3*-KO (Tian et al. 2015)	Severe weakness (heterozygotes normal)	Fast 2B fibre atrophy. Types 1 and 2A larger. More type 1 fibres in type 2B predominant muscles	Rods, thickened disrupted Z lines	(NR)	Internal nuclei
*Lmod3*^Δ2^-KO *Lmod3*^Δ10^-KO (Cenik et al. 2015)	Severe	Reduced myofibre size	Rods, disrupted sarcomeres	(NR)	Internal nuclei, accumulation of desmin, upregulation of sarcolipin
*KLHL40*-KO (Garg et al. 2014)	Severe/lethal	(NR)	Rods, thickened Z lines, Z-line streaming	> 50% reduction in hind limb strength	Complete disorganization in subset of fibres. Almost complete absence of *LMOD3*, nebulin 50% reduced. Increased expression of sarcomere genes. Klhl40 ± (heterozygous) mice also had reduced *LMOD3*, without growth defects or early lethality
*KLHL41*-KO (Ramirez-Martinez et al. 2017)	Severe/lethal (Het normal)	(NR)	Rods, Z-line streaming	(NR)	Aggregation and down-regulation of nebulin and only a slight decrease in *LMOD3* protein but not RNA, decrease in ssTNT and β-TPM

*NR* not reported, *ktr* rate of tension redevelopment

Many of the mouse models have shown early lethality, precluding their use as testbeds for experimental therapies, while others are less severely affected. An ideal model for the most common (mainstream or typical) form of nemaline myopathy caused by two different mutations in *NEB* is being developed (Laitila et al. submitted for publication). Mice devoid of nebulin, and showing early lethality, mirror the human disease only to a limited extent and do not consistently show rods (Bang et al. 2006; Witt et al. 2006). As no human patients with mutations causing total absence of nebulin have been reported, and since it has been thought that nebulin works as a ruler for thin filament length and maintaining Z-line structure (Ottenheijm et al. 2012), it is remarkable that the knock-out mice do form sarcomeres despite the absence of nebulin.

In a mouse model of *TPM3* nemaline myopathy (Gineste et al. 2014), the onset of muscle weakness, caused at least partially by hypotrophy of type 1 fibers, appeared to be delayed by compensatory hypertrophy of type 2 fibers, as in human patients.

In *Tnnt1* mice, depicting the “Amish” form of nemaline myopathy, there was severe weakness of the diaphragm (Wei et al. 2014), as in human patients, and an increase in fast 2B fibre types, but this myosin isoform is not expressed in human limb muscle.

A mouse model of *CFL*-*2* nemaline myopathy showed severe weakness, small body size and early lethality. Histologically, there was actin accumulation (as seen in humans with *CFL*-*2* mutations) whereas nemaline bodies were small and only seen on EM in severely disrupted fibres (Agrawal et al. 2012; Gurniak et al. 2014).

Mouse models of *LMOD3* nemaline myopathy showed atrophy of fibres with fast myosin, a 50% reduction of grip strength (Tian et al. 2015), small body size and normal lifespan (Cenik et al. 2015).

Mouse models of *KLHL40* and *KLHL41* nemaline myopathy showed early lethality (within days to weeks from birth). KLHL40-deficent mice had a secondary reduction in nebulin and *LMOD3* whereas the *KLHL41* knock-out mice only showed reduction of nebulin (Garg et al. 2014; Ramirez-Martinez et al. 2017). The rectangluar rods seen in human patients with nemaline myopathy caused by mutations in these two genes were not reported in the *Klhl40* model but they were apparent in the *Klhl41* knock-out mouse.

## Development of therapies for nemaline myopathies

There is currently no curative treatment for patients with nemaline myopathy, but much can be achieved by a multidisciplinary approach, addressing the management of symptoms and maintaining muscle strength, mobility, joint movements, and independence in the activities of daily living through exercise and physiotherapy. Particularly important is regular monitoring of respiratory function and addressing orthopaedic problems, especially any scoliosis (Wallgren-Pettersson et al. 2004; Wang et al. 2012).

Tyrosine as a treatment for nemaline myopathy has been advocated, but an apparently beneficial effect in a limited clinical trial of dietary tyrosine supplementation (Ryan et al. 2008) was not supported up by studies of the nemaline myopathy Tg*ACTA1*^D286G^ mouse model, nor the zebrafish model based on the same mutation (Messineo et al. 2018; Sztal et al. 2018a). Other amino acid supplements tested in zebrafish also showed no clear positive effect (Sztal et al. 2018a). The improvement in skeletal *Acta1* knock-out mice through upregulation of cardiac actin (Nowak et al. 2009) raises hopes for therapeutic implications for patients, but requires very early diagnosis. Another therapeutic option to explore is increasing the proportion of normal skeletal actin in heterozygous patients (Ravenscroft et al. 2011a, b). Interestingly, a zebrafish morpholino knock-down model of *ACTA1* nemaline myopathy showed a milder phenotype because of a transcriptional upregulation of an actin paralogue, i.e. through genetic compensation (Sztal et al. 2018b).

Experimental trials with myostatin in two mouse models of *Acta1* nemaline myopathy did not yield stronger mice, but in the Tg*ACTA1*^D286G^ mouse model the body size increased (Tinklenberg et al. 2018) and a similar trial in the *Acta1*^H40Y^ mouse model led to both larger size and longer life-span (Tinklenberg et al. 2016). The use of a myosin transgene to improve muscle function in an *Acta1* mouse model for nemaline myopathy (Lindqvist et al. 2016) and the use of calcium sensitizers to improve diaphragm function (Ochala 2010; Doorduin et al. 2012) raise interesting perspectives for the future.

## Conclusions

Our increasing understanding of the pathogenetic mechanisms, and the lines of therapeutic options explored hitherto make it timely to plan a natural history study of nemaline myopathy and an international patient registry. In doing so, the international collaborative effort will pave the way for therapeutic trials, once non-hazardous and potentially effective treatment modalities become available.
